# Polymeric Micelles, a Promising Drug Delivery System to Enhance Bioavailability of Poorly Water-Soluble Drugs

**DOI:** 10.1155/2013/340315

**Published:** 2013-06-27

**Authors:** Wei Xu, Peixue Ling, Tianmin Zhang

**Affiliations:** ^1^School of Pharmaceutical Science, Shandong University, Jinan 250012, China; ^2^Department of Pharmacy, Shandong Provincial Qian Foshan Hospital, Jinan 250014, China; ^3^Institute of Biopharmaceuticals of Shandong Province, Jinan 250101, China

## Abstract

Oral administration is the most commonly used and readily accepted form of drug delivery; however, it is find that many drugs are difficult to attain enough bioavailability when administered via this route. Polymeric micelles (PMs) can overcome some limitations of the oral delivery acting as carriers able to enhance drug absorption, by providing (1) protection of the loaded drug from the harsh environment of the GI tract, (2) release of the drug in a controlled manner at target sites, (3) prolongation of the residence time in the gut by mucoadhesion, and (4) inhibition of efflux pumps to improve the drug accumulation. To explain the mechanisms for enhancement of oral bioavailability, we discussed the special stability of PMs, the controlled release properties of pH-sensitive PMs, the prolongation of residence time with mucoadhesive PMs, and the P-gp inhibitors commonly used in PMs, respectively. The primary purpose of this paper is to illustrate the potential of PMs for delivery of poorly water-soluble drugs with bioavailability being well maintained.

## 1. Introduction 

Oral administration is the most commonly preferred route for drug delivery because of its simplicity, convenience, and patient acceptance, especially in the case of repeated dosing for chronic therapy [1–3]. In contrast to the intravenous administration, which probably results in toxic blood level after injection and sometimes an under concentration of the desired threshold towards the end of the dosing interval, oral chemotherapy can provide a prolonged and continuous exposure to a relatively lower and thus safer concentration [2]. Now, more than 60% of marketed drugs are used as oral products [4]. However, it is intricate to formulate a therapeutic agent for oral administration. The bioavailability of oral drugs is strongly influenced by two important parameters, solubility and permeability [3]. Based on that, the Biopharmaceutic Classification System (BCS) defines four categories of drugs [5]. Many existing and new therapeutic entities are characterized as BCS class II (low solubility and high permeability) or BCS class IV (low solubility and low permeability). Poorly water-soluble drug candidates encountered in drug discovery cause increasing problems of poor and variable bioavailability. It is estimated that approximately 70% of new chemical entities are poorly soluble in aqueous medium and many even in organic medium. Besides, approximately 40% of currently marketed immediate-release oral drugs are considered practically insoluble (solubility less than 100 *μ*g/mL) in water [6, 7]. Low solubility limits the drug dissolution rate, frequently resulting in low bioavailability of the oral drug [8]. To achieve the desired therapeutic concentration in the target sites, dose escalation study of the drug was often applied in clinic [9, 10]. However, it may be undesirable due to the possibility of increased toxicity and therefore decreased patient compliance. Meanwhile, the high drug loading of pharmaceutical products often makes it difficult to complete the study [11].

Nanotechnology brings some advantages to the drug delivery, particular for oral drug. It allows (1) the delivery of poorly water-soluble drugs; (2) the targeting of drugs to specific parts of the gastrointestinal tract (GI); (3) the transcytosis of drugs across the tight intestinal barrier; and (4) the intracellular and transcellular delivery of large macromolecules [12, 13]. In recent years, nanotechnology has been widely focused on by numbers of researchers throughout the world for its superiority in increasing efficacy, specificity, tolerability, and therapeutic index of corresponding drugs [14]. Several strategies have been proposed such as micronization, complexation, formation of solid solutions, microemulsification, and novel drug delivery systems, including nanoparticles, lipid-based vesicles, and micelles [15–18]. Among these approaches, polymeric micelles (PMs) have gained considerable attention in the last two decades as a multifunctional nanotechnology-based delivery system for poorly water-soluble drugs. The application of PMs as drug delivery system was pioneered by the group of H. Ringsdorf in 1984 [19] and subsequently used by Kataoka in the early 1990s through the development of doxorubicin-conjugated block copolymer micelles [20]. Due to their nanoscopic size, ability to solubilize hydrophobic drugs in large amounts and achieve site-specific delivery, PMs hold promise to obtain desirable biopharmaceutical and pharmacokinetic properties of drugs [21] and enhance their bioavailability. In this review article, we will discuss the development of the PMs and focus on the mechanisms of various kinds of PMs for enhancement of oral bioavailability. 

## 2. Absorption of Oral Drugs in the Gastrointestinal Tract

### 2.1. Pathways of Drug Absorption

**Figure 1:**
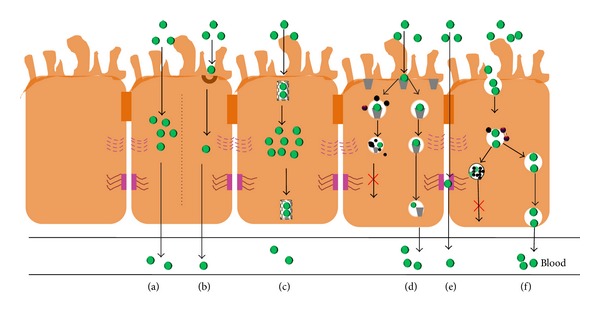
Schematic representation of the mechanisms involved in the absorption of exogenous drugs in the small intestine. (a) Transcellular transport; (b) active transport; (c) facilitated diffusion; (d) receptor-mediated endocytosis; (e) paracellular transport; (f) pinocytosis [3].

A drug that is administered orally must survive transit through the gastrointestinal (GI) tract. Although part of the absorption process occurs in the oral cavity and stomach due to the presence of salivary amylase and gastric protease (pepsin), the small intestine remains the major site for absorption [22]. There exist many pathways for nutrient absorption in the small intestine; however, the absorption of oral drugs is restricted to either transport through the cells (transcellular pathway, see Figure 1(a)) or between adjacent cells (paracellular pathway, see Figure 1(e)) [3]. Generally, the low-molecular weight hydrophobic entities which are able to diffuse through the membrane are absorbed by the transcellular pathway, and the absorption rate is determined by the concentration gradient across the intestinal membrane (Figure 1(c)). On the contrary, hydrophilic molecules cannot freely diffuse through the intestinal membrane, due to their low affinity for the lipidic constituents [23]. Therefore, in the absence of an appropriate membrane transporter, the paracellular pathway is the only available route for their absorption (Figure 1(e)). In some particular instances, drugs may be absorbed by fluid-phase endocytosis (pinocytosis), an energy-dependent saturable process in which the molecule travels inside membrane vesicles (Figure 1(f)).

### 2.2. Barriers for Absorption of Oral Drugs

Although oral administration is the preferred route for drug delivery, and the mechanisms of drug absorption have been widely studied, there still exists the serious problem of low bioavailability which has severely impeded the development of oral therapy. The bioavailability of a drug strongly depends on its intrinsic properties and physiological conditions. A drug that is administered orally must survive transit through the chemical and enzymatic GI liquids, cross the mucus layer and the epithelium before being absorbed [24, 25]. Intrinsic properties of drugs such as poor stability in the gastric environment, low mucosal permeability, and low solubility in the mucosal fluids will contribute to low absorption [26, 27]. Physiological factors such as gastrointestinal transit time, regional pH, surface area, enzymatic activity, and colonic microflora will also influence drug absorption [28]. 

Therefore, to achieve good absorption and bioavailability, oral drugs should be stable at the low gastric pH and have a reproducible and good pharmaceutical dissolution profile and adequate hydrophilic/lipophilic balance to cross the intestinal epithelial membrane. Furthermore, they should not induce significant gastrointestinal toxicities, such as nausea, vomiting, loss of appetite, or diarrhea, that would limit continued oral administration or result in poor compliance [29, 30]. To overcome these barriers and achieve above-mentioned requirements, several strategies have been proposed including the reduction of drug particle size [31], salt formation [32], or prodrug synthesis [33]. It is worth mentioning that nanosized carriers such as PMs [34] could encapsulate drugs into protective vehicles, avoiding destruction in the GI tract and releasing them in a temporally or spatially controlled manner, which could potentially enhance drug absorption and offer a promising direction for oral therapy [28]. 

## 3. Introduction of PMs

### 3.1. Formation of PMs

**Figure 2:**
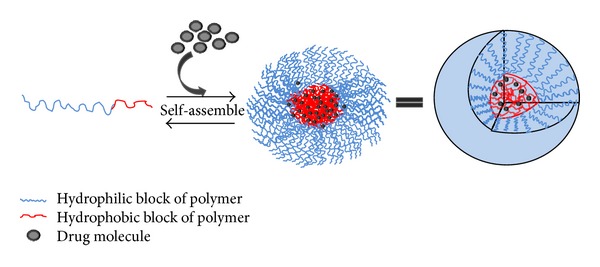
Formation and drug loading of PMs by self-assemble of amphiphilic block copolymers in aqueous solution.

PMs are self-assembled core-shell nanostructures formed in an aqueous solution consisting of amphiphilic block copolymers (see Figure 2) [35, 36]. Formation of micelles in aqueous solution occurs when the concentration of the block copolymer increases above a certain concentration named the critical aggregation concentration (CAC) or critical micelle concentration (CMC). At the CAC or CMC, hydrophobic segments of block copolymers start to associate to minimize the contact with water molecules, leading to the formation of a vesicular or core-shell micellar structure.

Theoretically, the formation of micelles is driven by decrease of free energy. The removal of hydrophobic fragments from the aqueous environment and the reestablishing of hydrogen bond network in water decrease free energy of the system and finally form the micelles. The typical methods used for encapsulation of poorly water-soluble drugs are dialysis method, oil-in-water emulsion solvent evaporation method, and solid dispersion method [37, 38]. Other methods used are direct dissolution [39], complexation [40], chemical conjugation [41], and various solvent evaporation procedures [42].

### 3.2. Structure of PMs

PMs present a great potential as a drug delivery system for compounds that are hydrophobic and exhibit poor bioavailability which results from the unique core-shell structure. The inner hydrophobic core enables incorporation of poorly water-soluble drugs thus improving their stability and bioavailability. Typically, the inner core of the PMs was formed with hydrophobic blocks of the copolymers by hydrophobic interaction. Besides, it can also be formed by electrostatic interactions, using charged block copolymers of oppositely charged macromolecules, resulting in the formation of polyion complex (PIC) micelles [43, 44]. In addition, there have been reports of PMs formed by complexation via hydrogen bonding [45–47] as well as metal-ligand coordination interactions [48], both referred to as noncovalently connected micelles. The outer shell of PMs was formed by the hydrophilic blocks of the copolymers, playing an important role in the in vivo behavior, particular for their steric stabilization and ability to interact with the cells [49]. Lengths of the hydrophobic and hydrophilic blocks affect the conformation of polymers in medium, as lengthier hydrophilic blocks of polymer cause it to remain monomeric in water [50].

Amphiphilic copolymers which constitute PMs are usually block copolymers [51, 52]. Block copolymers can be diblock copolymers or triblock copolymers. Generally, diblock copolymers of the A-B type, where A represents a hydrophilic block and B represents a hydrophobic block, are commonly used to design PMs, whereas triblock copolymers consist of two types of polymers (ABA) [53] or three types of polymers (ABC). Most drug carrier applications have been studied with A-B or A-B-A type block copolymers due to the close relationship between the properties of micelles and the structure of polymers [54]. The physicochemical characteristics of the building blocks influence the physical and biological properties of the PMs [55]. Hence, micelle-forming block copolymers have been the focus of several studies over the past few years. For oral drug delivery system, the block copolymers used to form micelles should (1) spontaneously self-assemble in water, (2) enhance drug solubility by several orders of magnitude and provide high loading efficiency, (3) remain stable upon dilution in the GI tract, (4) be biocompatible and nontoxic, and (5) be easy to synthesize at large scale [28, 56, 57]. The choice of core-forming polymers is the major determinant for important properties of PMs such as stability, drug loading capacity, and drug release profiles [58]. Poly(propylene oxide) (PPO) [53, 59] which belongs to Pluronics, poly(esters) such as poly(lactic acid) (PLA) [60], hydrophobic poly(amino acids) [61], copolymers of lactic acid and glycolic acids [62, 63], and poly(caprolactone) (PCL) [64], which are regarded as the commonly used core-forming blocks of PMs, and have been studied in the past 10 years. These core-forming polymers cover a wide range of structural diversity and polarity for solubilizing numbers of poorly water-soluble drugs [51, 52]. Meanwhile, the chemical nature and molecular weight of the hydrophilic block will strongly affect the stealth properties and accordingly influence the circulation kinetics of the micellar assembly. Poly(ethylene glycol) (PEG) is most commonly used as the hydrophilic segment of the block copolymers, since it is a nontoxic polymer with FDA approval as a component of various pharmaceutical formulations. Furthermore, its unique physicochemical properties (high water solubility, high flexibility, and large exclusion volume) provide good “stealth” properties for PMs [65, 66], while poly(N-vinyl-2-pyrrolidone) (PVP) [67] and poly(acrylic acid) (PAA) [68] are frequently used as PEG alternatives. 

## 4. PMs for Enhancement of Bioavailability

The main mechanisms involved in the enhancement of drug absorption by PMs are: (1) protection of the loaded drug from the harsh environment of the GI tract, (2) release of the loaded drug in a controlled manner at target sites, (3) prolongation of the residence time in the gut by mucoadhesion, and (4) inhibition of efflux pumps to improve drug accumulation [69]. Several physicochemical parameters seem to influence translocation of micelles across the epithelium, including surface hydrophobicity, polymer nature, and particle size [69]. There exist many characteristics of PMs that allow them to traverse across the epithelium. For example, PMs with appropriate particle size can be taken up and then cross the intestinal barrier [40, 70, 71]. Furthermore, to achieve a good bioavailability, drugs may be delivered at a specific region in the GI tract, the so-called absorption window. To reach the absorption window, PMs can be manipulated by coupling different types of polymers or by grafting various functional groups at the hydrophilic end of the copolymer, such as the pH-sensitive [72–74] and receptor sensitive groups [75]. 

### 4.1. Special Stability of PMs for Enhancement of Bioavailability

As we discussed above, GI tract is the major barrier for oral drugs. After oral administration, drugs will encounter the harsh physicochemical environment of the GI tract and be degraded due to the variation of pH levels as well as the presence of enzymes or bile salts. To ensure delivery of the carried drugs to the absorption sites, PMs must be able to resist rapid dissociation upon dilution and retain the stable core-shell structure before target sites. It is known that PMs possess two aspects of structural stability, thermodynamic and kinetic, provided by the entanglement of polymer chains in the inner core [76–78]. 

For a micelle to be thermodynamically stable, the copolymer concentration should be above its CMC. The CMC is influenced by the hydrophilic-lipophilic balance (HLB) of the block copolymer [79]. A reverse relationship between the CMC and hydrophobicity of the core-forming blocks has been shown in many studies: an increase in the hydrophobic block length results in a lower CMC if the hydrophilic segment is kept constant [80]. Generally, PMs show very low CMC values in a range from 10^−6^ to 10^−7^ M. These CMC values are much smaller than those of micelles formed from low-molecular weight surfactants (10^−3^–10^−4^ M) [81], which allows a series of dilution and still retain the micellar structure. The second aspect, kinetic stability of PMs, comes into the picture when the concentration of the copolymer falls below the CMC. Kinetic stability may be more important than the thermodynamic stability for the nonequilibrium drug delivery conditions. Unlike micelles formed from low molecular weight surfactant molecules, the kinetic stability of PMs is high for the stiff or bulky core structure. Therefore, the disassembly of PMs at a concentration below CMC occurs at a relatively slower rate because of the relatively high kinetic stability. The slow dissociation allows PMs to retain their integrity and drug content before reaching the target sites, which is also helpful to enhance oral bioavailability.

### 4.2. pH-Sensitive PMs for Enhancement of Bioavailability

It is indicated that non-pH-sensitive micelles may enhance drug solubilization but probably not necessarily the drug absorption. Free (readily absorbable) form of a drug is one of the most important requirements for absorption in the GI tract. However, drug release from such PMs will occur only by diffusion when polymer concentration is well above the CMC, preventing the complete drug release [11]. Moreover, Camilleri once studied the stomach emptying time (ca. 177 min) and the small bowel transit time (ca. 168 min) in healthy human volunteers by monitoring the migration of a radio-labeled marker previously mixed in their meal [82]. Thus, there is also a possibility that the PMs might be excreted before complete drug release or drug might not be released close to its absorption window in the GI tract. Several PMs systems designed to increase the oral bioavailability of hydrophobic compounds exhibit release times which largely exceeded the transit time in the small intestine [83, 84]. This is also true for surfactant micelles which have been found in some cases to impede the absorption of hydrophobic drugs due to excessive retention in the micellar phase [85]. Hence, when developing oral formulations for poorly water-soluble drugs, it is important to adequately control the release rate in order to avoid either precipitation upon dilution or sequestration within the micellar phase which may lead to incomplete absorption.

#### 4.2.1. Introduction of pH-Sensitive PMs

The potential disadvantage of normal PMs can be solved by application of additional stimuli that cause micelle destabilization in a specially controlled manner thus increasing the selectivity and efficiency of drug delivery to target sites. External factors such as heat [86, 87], light [88], and sound (ultrasound) [89, 90] have already been studied by many researchers. However, these external stimuli may only activate the carriers that are situated closely underneath the skin but not those deeply distributed in the body. The intracellular signals also play an important role in regulating drug release which causes a great deal of interests, and here we focus our attention on pH-responsive systems. 

As is known, blood and normal tissues have a pH of 7.23 [91]. The mildly acidic pH encountered in a tumor (pH *∼*6.8) as well as endosomes and lysosomes (pH 5.0–5.5) provides a potential trigger to accelerate the degradation of pH-sensitive PMs and release of encapsulated drugs. Therefore, numerous pH-sensitive polymeric micellar systems have been employed for intravenous administration of anticancer drugs to tumors [92–94]. In the GI tract, the pH varies from high acidity in the stomach (pH 1.0–2.5) to a neutral or slightly alkaline pH in the small intestine (pH 5.1–7.8) [95]. Such wide variation of pH along the GI tract has been utilized for controlled drug release from carriers [2]. Strategies to prevent GI degradation or to promote absorption in the intestine by making use of the pH gradient appear promising.

#### 4.2.2. Mechanisms of pH-Sensitive PMs for Enhancement of Bioavailability

**Figure 3:**
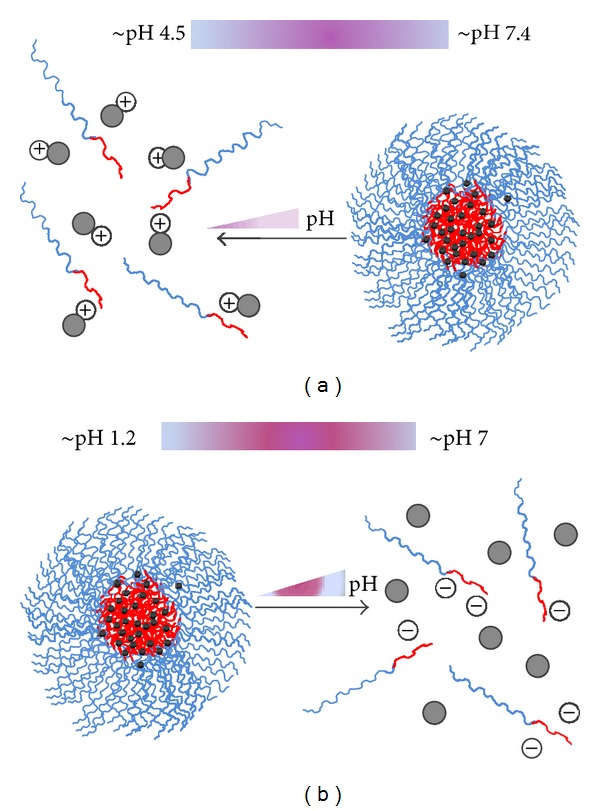
Schematic representation of the mechanisms of pH sensitivity. (a) PMs with basic core units, (b) PMs with acidic core units.

Among the various polymers composed micelles, polyacids, or polybases may be used as building blocks that impart pH sensitivity to drug release [73, 96]. Basic core monomeric units such as amines are uncharged and thus hydrophobic at high pH condition while hydrophilic upon protonation at low pH (see Figure 3(a)). On the contrary, acidic core units such as carboxylic acids are uncharged when protonated at low pH and become negatively charged at a relatively high pH (see Figure 3(b)). Many examples of “protonation” approaches to trigger destabilization of micelles have been reported, such as incorporating L-histidine [97, 98], pyridine, and tertiary amine groups [99] in their hydrophobic segments. PMs are formed at a pH above the pKa of the protonatable group, where the hydrophobic segment essentially is uncharged. As the pH decreases below the pKa, the ionization of the polymer causes increased hydrophilicity and electrostatic repulsions of the polymers, leading to the destabilization of the micelles and controlled drug release. 

#### 4.2.3. Polymers Commonly Used in Oral pH-Sensitive PMs

Acrylic-based polymers are widely used in oral pH-sensitive drug delivery, such as poly(methacrylic acid) (PMAA). PMAA retains a collapsed state in the low pH of the stomach and swells as it transits through the intestines. Blends of this polymer with polyethylacrylate (PMAA-PEA) and polymethacrylate (PMAA-PMA) can be tailored to dissolve in specific pH ranges corresponding to specific locations in the GI tract [100–102]. These pH-responsive micelles based on the acrylic acid core can be either multimolecular or unimolecular [103, 104]. Upon pH increase, the core of the unimolecular micelles became more polar hence promoting the release of the incorporated hydrophobic drug [103]. As these micelles do not possess a CMC, they have the advantage of being intrinsically stable upon dilution. Conversely to unimolecular micelles that maintain their integrity upon a change in pH, pH-sensitive multimolecular PMs based on ionizable polyanions disassemble following an increase in environmental pH. 

Kim and his coworkers hypothesized that the physical stability of hydrotropic polymeric (HP) micelles containing AA moieties may decrease in the intestine, releasing the loaded drugs faster in the intestine than in the stomach [105]. To examine this hypothesis, they took paclitaxel (PTX) as model drug and developed a hydrotropic polymer, PEG-b-(4-(2-vinylbenzyloxy)-N,N-(diethylnicotinamide)) (PEG-b-VBODENA), doped with AA units (≤50 mol%) to confer pH sensitivity to PMs, testing PTX loading/release profiles by changing the pH condition. They observed that the loading content and efficiency of PTX were governed by the pH of the loading medium, with both maxima at pH ≤ 4. Increasing the pH above the pKa of the polymers provoked a rapid dissociation of the complexes. The self-association into well-defined micellar structure is facilitated by the hydrophobic nonionizable Al(M)A units, whereas the pH sensitivity is conferred by the carboxylic acid groups of the MAA moieties [106]. The PTX release from HPC with morethan 20% AA contents was completed within 12 h in a simulated intestinal fluid (pH = 6.5) while the PMs without any AA moiety showed very slow release profiles. Therefore, the pH-dependent release of PTX from HP micelles can be used to increase the bioavailability of PTX upon oral delivery.

Some other groups have also developed the pH-sensitive oral drug delivery systems. In an earlier report, Sant et al. prepared and characterized a pH-sensitive PMs incorporating poorly water-soluble model drugs [104]. The self-assemblies were constructed from novel pH-sensitive polymers composed of poly(ethylene glycol)-block-poly(alkyl acrylate-co-methacrylic acid) (PEG-b-P(AlA-co-MAA)). Due to the presence of pendant carboxyl groups in the hydrophobic part, these copolymers exhibit pH-dependent aggregation behavior and form supramolecular micelles below pH 4.7. Hence, these copolymers dissociate partially or completely with increase in pH owing to the ionization of carboxylic groups. Two water-insoluble model drugs, named indomethacin (IND) and fenofibrate (FNB), were incorporated in the supramolecular assemblies by dialysis or oil-in-water (*O*/*W*) emulsion methods. The pH-dependent drug release in vitro from the micelles was also confirmed in their study. To make sure whether pH-sensitive PMs could improve the bioavailability of a poorly water-soluble drug, further in vivo study was carried out [1]. For in vivo study, FNB was chosen as the poorly water-soluble model drug. The pharmacokinetics of FNB incorporated in PMs was evaluated in male Sprague-Dawley rats after oral dosing and compared with the commercial micronized formulation, Lipidil MicroR and an FNB coarse suspension. The oral bioavailability of FNB from these self-assemblies revealed 156% and 15% increases versus FNB coarse suspension and Lipidil MicroR, respectively. The results suggest that these pH-sensitive PMs could efficiently improve the bioavailability of poorly water-soluble drugs. Other types of pH-controlled release carriers such as pH-sensitive polymer-drug conjugates [107, 108] are beyond the scope of PMs and not discussed in this review. 

### 4.3. Mucoadhesive PMs for Enhancement of Bioavailability

#### 4.3.1. Introduction of Mucoadhesive PMs

Nanocarriers for oral administration should adhere to mucus and cross the mucus layer. Drugs delivered to mucosal surfaces are usually efficiently removed by mucus clearance mechanisms [109]. The luminal surface of mucosal tissues is protected by a highly viscoelastic layer [110], and the protective coatings rapidly remove foreign particles from the GI tract which probably lead to low bioavailability. Unlike the relatively high requirements of intravenous infusions, oral formulations could include high-molecular weight polymers as long as these components are metabolizable and cannot find their way into the systemic circulation. Hence, it may be an effective means of increasing uptake of drugs with mucoadhesive PMs [111, 112], and there have been considerable interests in the concept of mucoadhesive PMs. Firstly, mucosal retention can be used to increase the transit time in the GI tract, resulting in prolonged time window for the release of the payload. Secondly, mucoadhesive polymers swell and fill the crevices of the mucous membrane, contributing to the effective surface area in contact with the intestinal mucosa and yielding a high local concentration of the drug [113]. Thirdly, bioadhesion could also localize the PMs at a given target site and increase the drug concentration gradient for the intense contact of the particles with the mucosal surface [27].

The ability to develop mucoadhesive interactions within the gut would be one of the key factors influencing their ability to promote oral absorption of the loaded drug. It was demonstrated that there exists a direct relationship between mucoadhesion and drug absorption [114, 115]. In fact, the development of adhesive interactions (between PMs and mucosa) would probably induce the immobilization of these carriers in intimate contact with the absorptive membrane. This fact would facilitate the establishment of a concentration gradient of the loaded drug from the PMs to the circulation, which finally results in an enhancement of absorption and bioavailability.

#### 4.3.2. Mechanisms of Mucoadhesive PMs for Enhancement of Bioavailability

Mucoadhesion is a complex phenomenon, and several steps have been suggested in mucoadhesive bond formation [116]. The first step is the spreading, wetting, and dissolution of mucoadhesive polymer at the interface. The second step is the mechanical or physical entanglement between the polymer and the tissue surface mucus layer, resulting in an interpenetration layer. The next step is the result of chemical interactions [116]. Mucoadhesion can be obtained by the building of either nonspecific interactions with the mucosal surface, such as covalent bonds, ionic bonds, hydrogen bonding, and van der Waals' interactions [117], or specific interactions by functionalizing polymers with targeting ligands (e.g., lectins [118, 119]) or reactive groups such as thiols [120]. 

The fates of the mucoadhesive PMs in the GI tract include at least three different pathways: mucoadhesion, translocation through the mucosa or transit, and direct faecal elimination. Among the various factors, the surface charges of PMs seem to play an important role in particle uptake. On one hand, the negatively charged intestinal mucosa, due to the existence of glycocalyx, attracts more positively charged PMs. Therefore, a considerable number of studies have been conducted using positively charged polymers such as chitosan to increase residence time in the GI tract [121, 122]. On the other hand, the particle mobility also seems to be strongly dependent on surface charges, and it was indicated that transport rates were inversely related to particle surface potentials. Negatively charged particles display significantly higher transport rates than near neutral or positively charged particles whose transport was probably limited by particle aggregation and electrostatic adhesive interactions with mucosa [123]. Crater and Carrier demonstrated a 20–30 times faster diffusion for anionic particles in comparison with cationic ones [123], which proved the opinion discussed above. Therefore, it is crucial to control the balance between mucoadhesion and mucus penetration for an efficient oral delivery. 

#### 4.3.3. Polymers Commonly Used in Mucoadhesive PMs

Polymers such as cross-linked polyacrylic acids (PAA) [124–126], carboxypolymethylene, carboxymethyl cellulose, alginate, chitosan (CS), and their derivatives [127–129] are commonly accepted as mucoadhesive and safe polymers. Mucoadhesive polymers, especially positively charged polymers, were preferential to enhance drug absorption by prolonging the residence time at the site of absorption. Chitosan (CS), a linear amino polysaccharide composed of randomly distributed (1–4) linked d-glucosamine and N-acetyl-d-glucosamine units, is a well-known naturally occurring cationic biopolymer, which has received increasing attention owed to its biocompatibility, nontoxicity, and low immunogenicity [130, 131]. The adhesive properties of chitosan caused by the development of electrostatic interactions with glycoproteins of mucus [132] are of primary interest for oral delivery and its cationic properties below pH 6.5 favor the mucoadhesive ability. Moreover, among the existing cationic polymers, chitosan is an ideal candidate for oral DNA and protein delivery [133] due to its mucoadhesive properties and its ability to induce a transient opening of the tight junctions [134]. Nevertheless, due to the insolubility of chitosan observed at pH values above its pKa (6.4) in water, micelles of amphiphilic chitosan rapidly precipitate in biological solution (pH 7.4). Therefore, water-soluble chitosan derivatives have often been used for development of drug delivery systems like glycol chitosan (GC) and chitosan oligosaccharide (CSO), exhibiting good solubility over a broad range of pH [135, 136]. 

Other synthetic mucoadhesive polymers have been currently investigated in pharmaceutical formulations including PEG, cellulose derivatives (methylcellulose) [137, 138] and hydroxypropyl cellulose (HPC) [139], and polyelectrolytes (PAA) [39]. These polymers bind to the mucus via noncovalent bonds such as hydrogen bonding, electrostatic interactions, and van der Waals forces. Mucus interpenetration and chain entanglement may also contribute to the phenomenon of mucoadhesion, particularly with regard to uncharged polymers. Another commonly used mucoadhesive polymers are Pluronic-PAA copolymers. Strong mucoadhesive properties of the Pluronic-PAA copolymers originate from both the carboxyl-mucin interactions and the ability of the polyether segments to interpenetrate into and anchor the copolymer on the mucosa [124]. Mucoadhesive parameters of several types of Pluronic-PAA copolymers have already exceeded those of Carbopol or carbomer (lightly cross-linked PAA), which is an industry standard for mucoadhesive polymers used as pharmaceutical excipients. According to previous studies, mucoadhesive PAA and thiomers increase the residence time of insulin at the site of intestinal absorptive cells, thus enhancing its intestinal absorption [140–142]. Investigators assumed that the insulin uptake can be significantly enhanced after oral administration due to the positive attributes of the thiomer PAA-Cys including mucoadhesion, permeation enhancement and shielding against enzymatic degradation. 

Much stronger bioadhesion can be achieved by functionalizing polymers with targeting ligands (e.g., lectins) [118, 119] or reactive groups such as thiols [120]. Lectins are proteins or glycoproteins of nonimmunological origin which specifically recognize sugar molecules and therefore are capable of binding to glycosylated membrane components [143, 144]. Sugars are all present in glycoproteins and glycolipids of mammalian mucosa, either at the surface of epithelial cells or in mucous layers. Through strong adherence to glycoproteins and glycolipids in the membrane of enterocytes, lectins may prove useful in both prolonging the transit time of a host cargo through the small intestine as well as promoting its uptake via receptor-mediated endocytosis. Bernkop-Schnürch and coworkers have demonstrated that the thiolation of classical PMs substantially increases their mucoadhesive properties and therefore further improves the oral absorption of therapeutic proteins [145]. Surface-exposed thiols are thought to form disulfide bonds with cysteine-rich subdomains of mucus glycoproteins. Thiolated polymers also exhibit an increased permeation-enhancing effect as well as enzyme inhibitory properties [145]. Thiol-decorated polyion complex micelles prepared through complexation between PEG-*b*-poly(2-(N,N-dimethylamino)ethyl methacrylate) and a 20-mer oligonucleotide have been shown to interact with mucin through the formation of disulfide bonds [146]. While these micelles were initially designed to carry nucleic acid drugs, a similar strategy may be applied to deliver hydrophobic drugs through the use of thiol-functionalized PEG-b-PLA or PEG-b-PCL PMs [147].

### 4.4. P-gp Inhibitors for Enhancement of Bioavailability

#### 4.4.1. Introduction of P-gp

Besides uptake, drugs are often pumped out of enterocytes by efflux transporters on the surface of intestinal mucosa. The extent of absorption for poorly water-soluble drugs (and orally administered drugs in general) is affected by these efflux pathways [148]. Among the efflux transporters, the most well known and widely studied is the P-glycoprotein (P-gp) efflux transporters [149]. Pgp is a 170-kDa membrane transporter which is part of the ATP-binding cassette (ABC) [150]. Using ATP, the human multidrug resistance-associated protein (MDR1) and P-gp can actively transport a wide range of relatively hydrophobic, amphipathic drugs out of the cell. When drugs encapsulated in PMs, they remain mainly associated with the particles and are not likely to be substrate of the efflux pumps. However, hydrophobic drugs can be released from the micelles and are more likely to be transported by the efflux pumps [151]. Compounds transported by P-gp include important anticancer drugs like Vinca alkaloids [152], anthracyclines [153], epipodophyllotoxins, and taxanes [154]. So ABC transporters may reduce the amount of drug absorbed and limit bioavailability in a dose-dependent, inhibitable, and saturable manner [155]. Due to its ability to expel therapeutics, the presence of intestinal P-gp is associated with a decrease in oral bioavailability and is thought to be one of the most significant causes for decreased permeability and therefore oral bioavailability. Therefore, modulation of its activity is regarded as a potential means to improve drug bioavailability.

#### 4.4.2. Polymers Commonly Used in P-gp Inhibiting PMs for Enhancement of Bioavailability

The first P-gp inhibitors proposed were substrates that could bind to the protein and inhibit its activity. Several drugs, including cyclosporine A (cyA) and verapamil, have been studied for this purpose [156, 157]. However, these molecules may be associated with toxic side effects, and amphiphilic polymers were presented as a potential alternative [158]. Mostly, the inhibition of efflux transport with amphiphilic polymers appears to be related to a modification of the fluidity of the cellular membrane [159]. This inhibitory effect has been demonstrated with both low-molecular weight and polymeric micelles, among which D-a-tocopheryl polyethylene glycol succinate (TPGS) [160, 161] and Pluronics have been extensively studied.

**Figure 4:**
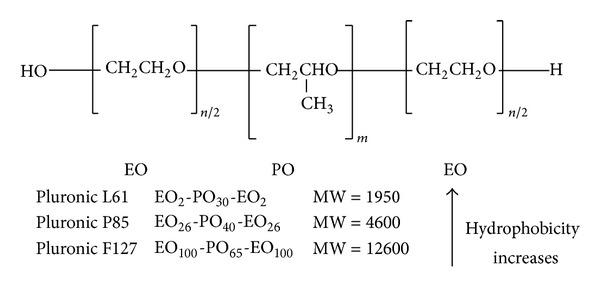
Pluronic block copolymers available from BASF (Wyandotte, MI, USA) contain two hydrophilic EO blocks and a hydrophobic PO block [167].

Pluronic block copolymers (also known under their nonproprietary name “poloxamers”) consist of hydrophilic ethylene oxide (EO) and hydrophobic propylene oxide (PO) blocks arranged in a basic A-B-A structure: EO_*n*/2_-PO_*m*_-EO_*n*/2_. The structure formula of Pluronic block copolymers is shown in Figure 4. Membrane fluidization is known to contribute to inhibition of P-gp efflux function. Pluronic block copolymers are known to induce drastic changes in the microviscosity of cell membranes, and these changes can be attributed to the alterations in the structure of the lipid bilayers as a result of absorption of the block copolymer molecules on the membranes [162]. Yoncheva et al. once prepared, characterized, and evaluated the pharmacokinetics of PTX incorporated in stabilized Pluronic micelles [49]. The stabilization of micelles was performed by cross-linking of their core, aiming to prevent disaggregation of micelles upon dilution in physiological fluids. Moreover, Pluronic copolymers may inhibit the activity of drug efflux transporters such as P-gp, MRPs, and BCRP [163, 164], which make it an adequate strategy to increase the bioavailability and promote the efficacy of PTX. Furthermore, it is believed that inhibition of P-gp ATPase activity, presumably through nonspecific changes in lipid and protein conformation and mobility, has a major contribution to the inhibition of P-gp efflux function [3]. Pluronic copolymers could inhibit drug efflux transporters, drug sequestration in acidic vesicles, and the glutathione/glutathione S-transferase detoxification system in an energy-dependent manner. Therefore, ATP depletion caused by the inhibition of the ATPase activity induced by the Pluronic copolymers has been proposed to be a reason for chemosensitization of these cells [165, 166].

**Figure 5:**
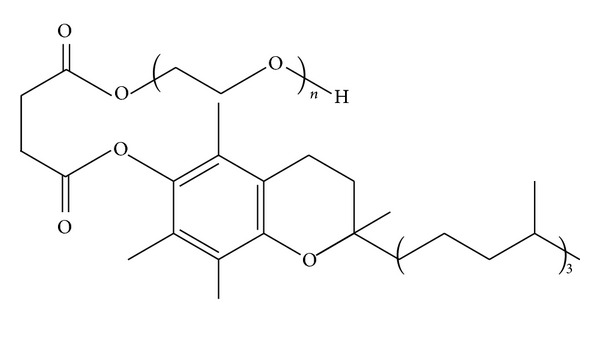
Structure of D-a-tocopheryl polyethylene glycol succinate (TPGS).

D-a-tocopheryl polyethylene glycol succinate (Vitamin E TPGS or simply TPGS) (see Figure 5) is a water-soluble derivative of natural Vitamin E, which is formed by esterification of Vitamin E succinate with polyethylene glycol (PEG) [168]. Therefore, it has advantages over PEG and Vitamin E in application of various drug delivery device, including extending the half-life of the drug in plasma and enhancing the cellular uptake [169]. TPGS has amphiphilic structure of lipophilic alkyl tail and hydrophilic polar head with an HLB value of 13.2 and a low CMC value [170]. 

The effect of TPGS on the bioavailability of a P-gp substrate was first reported in enhancing CyA absorption. It was initially postulated that the improvement in oral availability was due solely to micelle formation and increased drug solubility. Subsequently, Chang and coworkers demonstrated an increased CyA absorption at TPGS concentrations below the CMC [171]. Since CyA is a known P-gp substrate, the authors hinted at a possible mechanism implicating the efflux transporter, a premise which was later confirmed. Dabholkar and his coworkers made use of PEG-PE/TPGS mixed micelles as drug carrier and investigated some properties of the efficiency in solubilizing PTX and the ability to bypass the P-gp-mediated drug efflux [172]. It was shown that PTX was efficiently solubilized in the nontoxic PEG-PE/TPGS micelles, and the entrapment was quite stable with only about 20% of the incorporated drug released from micelles after 48 h at 37°C. In addition, PTX-containing PEG-PE/TPGS micelles were stable in vitro under various conditions, in particular, at low pH values and in the presence of bile acids, which is especially important for oral administration. Contrary to other surfactants, TPGS seems to have only a minor effect on membrane fluidity [173], challenging earlier reports [159]. Indeed, it was speculated that the inhibition of P-gp resulted from a decrease in ATPase activity following substrate binding [173]. Further in vitro studies were carried out to investigate the mechanisms of P-gp inhibition using Caco-2 cells model [174]. The data suggest that TPGS is neither a P-gp substrate nor a trigger of intracellular ATP depletion. Instead, TPGS might act as an allosteric modulator not involving the Cis(Z)-flupentixol binding site. 

In addition, some other amphiphilic polymers have been reported as P-gp inhibitors, such as mPEG-block-polycaprolactone [175], PEG-phosphatidylethanolamine [172], PEG-b-PLA [176], mPEG-poly(caprolactone-trimethylene carbonate) [177], and N-octyl-O-sulfate chitosan [178]. Among them, N-octyl-O-sulfate chitosan (NOSC) has been extensively studied. NOSC, synthesized by Q. Ping's group, is an amphipathic chitosan derivative, carrying sulfated groups as hydrophilic moieties and octyl groups as hydrophobic moieties [179]. The oral bioavailability of PTX-loaded NOSC micelles and Taxol was further compared. It was suggested that NOSC, an inhibitor of P-gp, could enhance the oral absorption of PTX by a P-gp-independent micelle internalization [180]. In vivo study, the oral bioavailability of PTX loaded in NOSC micelles was increased by 6-fold in comparison with that of an orally dosed Taxol. In the Caco-2 cell uptake studies, NOSC micelles brought about a significantly higher amount of PTX accumulated via both clathrin- and caveolae-mediated endocytosis. The mechanism of P-gp inhibition by NOSC was probably related to interfering with the P-gp ATPase rather than reducing the P-gp expression. 

## 5. Conclusion

Oral administration is the most commonly preferred route for drug delivery, especially in the case of repeated dosing for chronic therapy. To achieve good oral absorption of poorly water-soluble drugs, the loaded drug should be protected from the harsh gastrointestinal environment and release in a controlled manner at the target sites. In this review article, we aim to illustrate the potential of PMs for delivery of poorly water-soluble drugs, especially in the areas of oral delivery. It was suggested that PMs could enhance the oral drug bioavailability probably because the special stability (thermodynamic and kinetic stability) facilitating the safe transport of PMs through the GI tract, the pH-sensitivity of PMs promoting the controlled release properties of loaded drugs at target region, the mucoadhesivity of PMs prolonging the residence time in the gut, and the P-gp inhibitors contributing to drug accumulation. To make a methodical layout, we introduced various kinds of PMs separately in this article. However, a possible direction of combining two or more properties, such as pH-sensitive and mucoadhesive PMs, has gained much attention and offers a promising way to enhance the bioavailability of oral delivery.
